# Lin^neg^ Sca-1^high^ CD49f^high^ prostate cancer cells derived from the Hi-Myc mouse model are tumor-initiating cells with basal-epithelial characteristics and differentiation potential *in vitro* and *in vivo*

**DOI:** 10.18632/oncotarget.7535

**Published:** 2016-02-20

**Authors:** Achinto Saha, Jorge Blando, Irina Fernandez, Kaoru Kiguchi, John DiGiovanni

**Affiliations:** ^1^ Division of Pharmacology and Toxicology, College of Pharmacy, The University of Texas at Austin, Austin, TX 78723, USA; ^2^ Dell Pediatric Research Institute, The University of Texas at Austin, Austin, TX 78723, USA; ^3^ Stem Cell Transplantation Department, MD Anderson Cancer Center, The University of Texas, Houston, TX 77030, USA

**Keywords:** HMVP2 Cells, Hi-Myc mice, cancer stem cells, EMT, ventral prostate

## Abstract

A cell line was established from ventral prostate (VP) tumors of one-year-old Hi-Myc mice. These cells, called HMVP2 cells, are Lin^neg^Sca-1^high^CD49f^high^ with high CD44 and CD29 expression and express CK14, Sca-1 and CD49f (but not CK8), suggesting basal-epithelial characteristics. Furthermore, HMVP2 cells form spheroids and both the cells and spheroids produce tumors in syngeneic mice. After four days of culture, HMVP2 spheroids underwent a gradual transition from Lin^neg^Sca-1^high^CD49f^high^ expression to Lin^neg^Sca-1^low^CD49f^low^ while a subpopulation of the cells retained the original Lin^neg^Sca-1^high^CD49f^high^ expression pattern. Additional cell subpopulations expressing Lin positive markers were also present suggesting further differentiation of HMVP2 spheroids. Two additional highly tumorigenic cell lines (HMVP2A1 and HMVP2A2) were isolated from HMVP2 cells after subsequent tumor formation in FVB/N mice. Concurrently, we also established cell lines from the VP of 6 months old Hi-Myc mice (named as HMVP1) and FVB/N mice (called NMVP) having less aggressive growth properties compared to the other three cell lines. AR expression was reduced in HMVP2 cells compared to NMVP and HMVP1 cells and almost absent in HMVP2A1 and HMVP2A2 cells. These cell lines will provide valuable tools for further mechanistic studies as well as preclinical studies to evaluate preventive and/or therapeutic agents for prostate cancer.

## INTRODUCTION

Solid tumors contain a population of cells that can self-renew referred to as tumor-initiating cells, or cancer stem cells (CSCs), that can give rise to tumors in an appropriate host [1–6]. Current evidence suggests that these cells are a key component for tumor development, progression and metastases, and even recurrence after surgical or chemical removal of tumors [7–11]. Human and mouse prostate contains a pseudo-stratified epithelium with three types of differentiated epithelial cells: luminal, basal, and neuroendocrine [12]. Luminal epithelial cells are the major component of normal prostate and prostate cancer (PCa). These are cuboidal to columnar cells that produce secreted proteins and also express characteristic markers such as cytokeratin (CK) 8, CK18, NKX3.1, and high levels of androgen receptor (AR) [12]. Basal cells, which are located underneath the luminal epithelium and adhere strongly to basement membrane (BM) primarily express p63, CK5, and CK14 [12]. Finally, neuroendocrine cells express markers such as chromogranin A and synaptophysin, although their exact functions remain unclear [12]. PCa stem cells (PCSCs) are epithelial stem-like cells with the potential for initiating prostate tumors [13–15]. However, whether the PCSC populations in mice have a luminal or basal phenotypes is still uncertain and there is evidence to support both of these hypotheses [16].

Recently, several groups have isolated PCSCs using a mixed combination of cell markers from both human and mouse tissue [2, 5, 17–21]. Using cells isolated from prostate glands from the cPTEN^−/−^ L mouse model, Liao et. al. were able to identify a stem cell subpopulation exhibiting lineage negative (Lin^neg^), Sca-1 positive (Sca-1^pos^) and CD49f positive (CD49f^pos^) marker expression [21]. This subpopulation showed the capacity to generate spheroid-forming structures when cultivated in Matrigel and an increased proliferation rate when co-cultivated with cancer-associated fibroblast [18, 21]. Lawson et. al. showed that sorting prostatic cells for Lin^neg^ Sca-1^pos^ CD49f^pos^ expression results in a 60-fold enrichment for colony and sphere-forming cells that can self-renew and expand to form spheres for many generations [3]. Lin^neg^ Sca-1^pos^ CD49f^high^ cells derived from the prostate were capable of forming spheres in 3D cultures, eventually promoting the progression to prostate carcinoma [20]. Finally, Watson et. al. isolated and characterized a tumorigenic cell line (Myc-CaP cells) from Hi-Myc mice and reported their growth properties and stem cell phenotypic characteristics [22]. They also showed amplified androgen receptor gene expression as well as androgen dependent growth of this cell line in soft agar and in mice.

In the current study, we used the Hi-Myc transgenic mouse model for isolation of putative PCSCs from prostate tumors. In this mouse model, over-expression of c-Myc in the prostate is directed via the ARR_2_Pb probasin promoter [23]. Prostatic epithelial expression of c-Myc in the dorsolateral prostate (DLP), ventral prostate (VP), and anterior prostate (AP) lobes results in complete penetrance of prostatic intraepithelial neoplasia (PIN) as early as 2 to 4 weeks of age, which progress to locally invasive adenocarcinomas within 6 to 12 months of age [23, 24]. A population of cells possessing PCSCs characteristics both *in vitro* and *in vivo* was isolated from tumors of the VP that developed in one-year old Hi-Myc mice. These cells called ‘Hi-Myc Ventral Prostate 2 cells’ (HMVP2 cells) exhibited stem-like characteristics such as sphere-formation and sphere re-generation and produced tumors when injected into syngeneic hosts. In addition, HMVP2 cells expressed unique markers previously shown to be associated with PCSCs. Moreover, HMVP2 cells were able to differentiate to mixed sub-populations when grown as spheroids and in allograft tumors. Several other cell lines were also generated as part of this study, including a cell line from wild-type FVB/N mice (referred to as Normal Mouse Ventral Prostate; NMVP cells). These cell lines will provide useful tools for future mechanistic studies as well as preclinical studies with potential chemopreventive and/or therapeutic agents for PCa.

## RESULTS

### Establishment of cell lines

**Figure 1:**
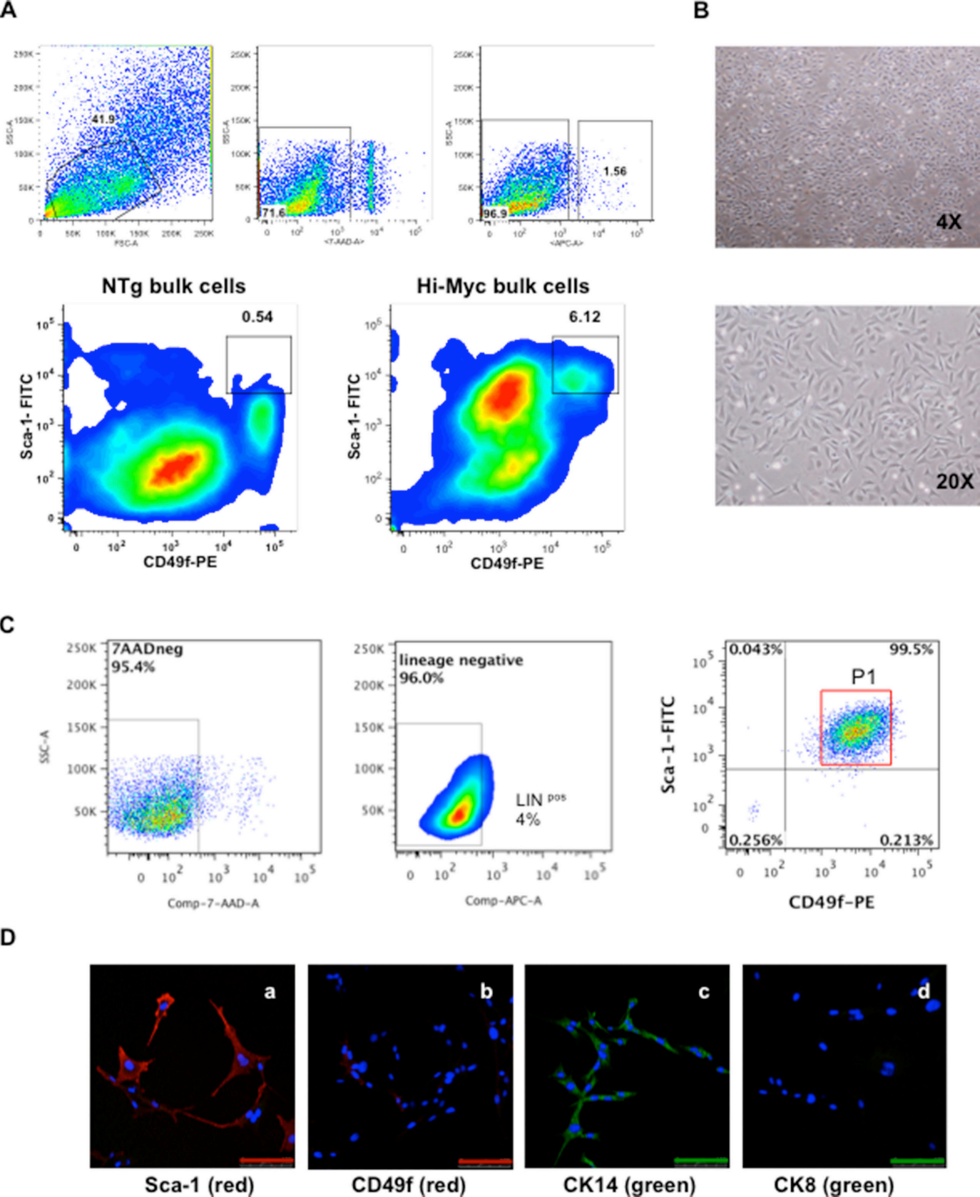
Isolation and characterization of HMVP2 cells (**A**) Representative FC analyses from both bulk cells isolated from ventral prostate glands of NTg mice (NTg bulk cells, bottom left) and tumoral prostate glands from Hi-Myc transgenic mice (Hi-Myc bulk cells, bottom right) (all cells isolated from one year old animals). FC analyses shows increased numbers of cells expressing Lin^neg^ Sca-1^high^ CD49f^high^ markers from the transgenic group (6.12%) compared to NTg animals (0.54%). [Lineage negative (APC), Sca-1 (FITC), CD49f (PE) and 7AAD (dead cells exclusion marker)]. (**B**) HMVP2 cells in culture at low (4×) and higher (20×) resolution. HMVP2 cells have a triangular shaped cytoplasm and a large rounded nucleus. (**C**) FC analyses from HMVP2 cells expressing Lin^neg^ Sca-1^high^ CD49f^high^ cells (P1). (**D**) IF staining of HMVP2 cells for Sca-1 (**a**), CD49f markers (**b**) CK14 (**c**), and CK8 (**d**). (The length of bar in Panels a-d is 100 μm).

Cells isolated from the VP of mice were screened by flow cytometry (FC) analyses for a series of markers associated with CSCs in various types of tumors [1, 5, 17, 19–21]. First, cells derived from the VP of both one year old FVB/N non-transgenic (NTg) littermate control and Hi-Myc mice, were screened for the Sca-1 and CD49f markers gated on the lineage negative population. Bulk cells derived from NTg littermates showed a high number of cells expressing low Sca-1 and CD49f when gated in Lin^neg^ cells, (i.e., Lin^neg^ Sca-1^low^ CD49f^low^)(Figure 1A) with a small number of cells exhibiting high expression of Sca-1 and CD49f (i.e., Lin^neg^ Sca-1^high^ CD49f^high^). Cells isolated from the VP of Hi-Myc mice (Hi-Myc bulk cells) showed populations with both high and low positive expression for Sca-1 and CD49f markers when gated on lineage negative cells (i.e., Lin^neg^ Sca-1^high^ CD49f^high^ and Lin^neg^ Sca-1^low^ CD49f^low^). Cells from both NTg and Hi-Myc mice showed lineage positive cells (Lin^pos^) (Figure 1A). Lin^neg^ excludes erythroid cells (Ter119), hematopoietic cells (CD45) and endothelial cells (CD31) [2].

In a separate experiment, cells isolated from the VP of one year old Hi-Myc mice were seeded in petri dishes with RPMI medium (with 10% FBS) and cultured continuously. After 10 passages, a homogenous population of small triangular shaped cells was established (Figure 1B). These cells were named Hi-Myc Ventral Prostate 2 or HMVP2 cells. FC analyses from the HMVP2 cells (10 passages) showed a high number of cells expressing Lin^neg^ Sca-1^high^ CD49f^high^ (P1, 95.5%) and a significantly lower number of Lin^pos^ cells (4%) (Figure 1C) compared to the original bulk Hi-Myc cells derived from the VP glands of one-year-old mice. Immunofluorescence (IF) staining of the HMVP2 cells also showed positive expression of both Sca-1 and CD49f (Figure 1D). In addition, HMVP2 cells were positive for CK14, and showed negative expression of CK8 (Figure 1D). Based on these marker analyses, HMVP2 cells appeared to have retained basal-epithelial characteristics.

### Analysis of spheroid-formation and cell differentiation

**Figure 2:**
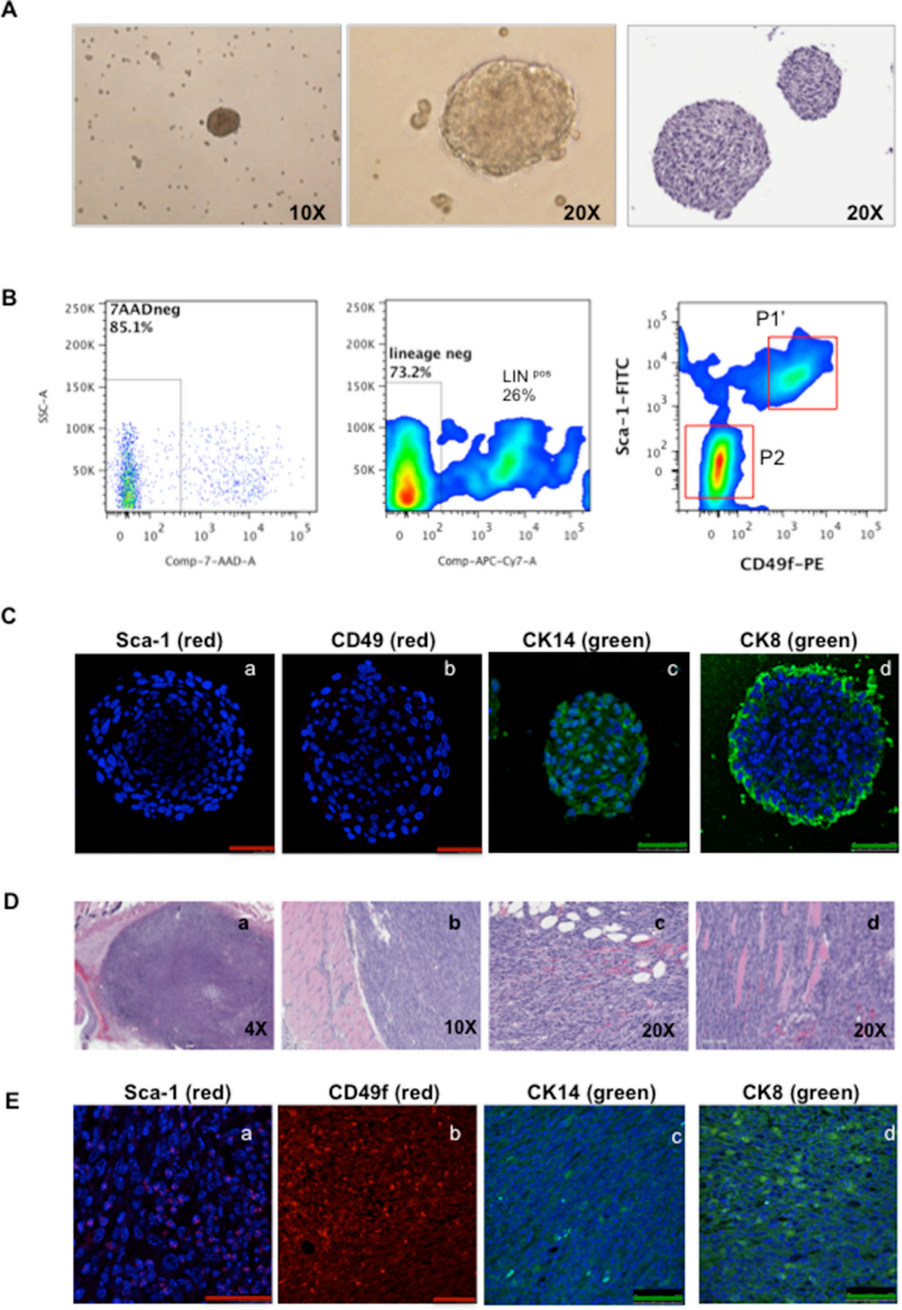
Characterization of spheroids and tumors derived from HMVP2 spheroids (**A**) HMVP2 spheroids after four days of culture (appearance of spheroids in culture, left (10×) and middle (20×) panels; H & E staining of a representative HMVP2 spheroid, right panel). (**B**) FC analyses from HMVP2 spheroids after four days of culture. HMVP2 spheroids express at least three different cell subpopulations, i.e; two lineage negative subpopulations: Lin^neg^ Sca-1^high^ CD49f^high^ (P1′), Lin^neg^ Sca-1^low^ CD49f^low^ cells (P2) and Lineage positive (Lin^pos^) cells. (**C**) IF of the HMVP2 spheroids showing a light positive staining for Sca-1 (**a**) and CD49f (**b**), and positive expression for CK14 (**c**) and CK8 (**d**). (The length of bar in Panels a-d is 50 μm). (**D**) Representative H & E stained sections of tumors derived from HMVP2 spheroids at low and higher magnification (a-d). Allograft tumors can be seen as undifferentiated masses containing a variety of cell types including epithelial-shaped cells, blood vessels, inflammatory cell and adipocyte infiltration.(**E**) Allograft tumors from HMVP2 cell spheroids showed positive staining for Sca-1 (**a**) CD49f (**b**) CK14 (**c**) and CK8 (**d**). (The length of bar in Panels a-d is 50 μm).

HMVP2 cells were able to generate spheroid structures when cultured in ultralow adherent dishes (Figure 2A). Cells harvested from spheroids displayed two new sub-populations in addition to the original Lin^neg^ Sca-1^high^ CD49f^high^ (Figure 2B, P1′) including a group of cells expressing Lin^pos^ markers and another group expressing Lin^neg^ Sca-1^low^ CD49f^low^ markers (Figure 2B, P2). Additionally, IF staining for Sca-1 and CD49f markers in spheroids showed reduced expression compared to cultured HMVP2 cells (compare Figure 2C with Figure 1D). HMVP2 spheroids were positive for CK14 and also showed positive expression of CK8 (CK8 was expressed at a low level in monolayer cells). These data suggested that HMVP2 cells differentiated from single layer cells with a basal-epithelial phenotype to a more luminal-epithelial cell phenotype during spheroid formation.

To further analyze this possibility, we conducted a time course experiment. HMVP2 cells were seeded for spheroid formation and cells were stained for FC analyses at 0, 24, 48, 72 and 96 hours (Supplementary Figure S1). At the time of seeding, a single homogenous cell type expressing only Lin^neg^ Sca-1^high^ CD49f^high^ (P1) markers was detected (Supplementary Figure S1A, 0 hr.). Spheroids showed a gradual shift to at least three cell subpopulations, including, Lin^pos^ cells, Lin^neg^ Sca-1^high^ CD49f^high^ cells (P1′) and Lin^neg^ Sca-1^low^ CD49f^low^ cells (P2) (Supplementary Figure S1A, 24 to 96 hrs and Supplementary Figures S1B and S1C). In order to further characterize the HMVP2 cells and spheroids, we performed additional staining for a set of markers previously described by others to be associated with CSCs, including CD34, CD44 and CD29 [9, 25–30]. This new set of markers was gated in both Lin^neg^ Sca-1^high^ CD49f^high^ and Lin^neg^ Sca-1^low^ CD49f^low^ cells. As shown in Supplementary Figure S1D, CD34 was absent in both the Lin^neg^ Sca-1^high^ CD49f^high^ cells at 0 hr, and in the more differentiated LIN^neg^ Sca-1^low^ CD49f^low^ cells at 24 hrs, although CD34 expression gradually started to increase in both cell sub-populations up to ~ 40–50% after 96 hrs in culture. This observation suggested differentiation from progenitor type cells to a more endothelial/hematopoietic cell population (Supplementary Figure S1D left column and Supplementary Figure S1E). CD44 was initially expressed in a large proportion of the original HMVP-2 Lin^neg^ Sca-1^high^ CD49f^high^ cells (~ 90%) and the expression was lower in the Lin^neg^ Sca-1^low^ CD49f^low^ sub-population after one day of culture (~ 30%). CD44 was gradually decreased in the cells expressing LIN^neg^/SCA-1^high^/CD49f^high^ to approximately 60% at 96 hrs suggesting a loss of pluripotency. CD44 expression was almost completely absent in the Lin^neg^ Sca-1^low^ CD49f^low^ expressing cells, indicating further cell differentiation (Supplementary Figure S1D middle column and Supplementary Figure S1F). CD29 was highly expressed in the Lin^neg^ Sca-1^high^ CD49f^high^ cells throughout the four day assay and showed a significant reduction on the Lin^neg^ Sca-1^low^ CD49f^low^ sub-population further indicating differentiation of the original population (Supplementary Figure S1D right column and Supplementary Figure S1G). Collectively these data suggest that the original HMVP2 Lin^neg^ Sca-1^high^ CD49f^high^ cells have the capability to generate several cell sub-types, from basal-epithelial to luminal-epithelial as well as other cell types. However, more functional assays need to be performed in order to fully validate this initial characterization.

Lastly, in order to look at self-renewal ability of the HMVP2 cell line, single cell suspensions were seeded in ultra-low culture dishes for spheroid formation. After four days of culture, cells were dissociated and re-seeded in new dishes for re-regeneration of spheroids. This experiment was repeated 15 times without any alteration of the cell growth capability or loss of the ability to generate subsequent spheroids (data not shown).

### HMVP2 cells are tumor-initiating cells

HMVP2 cells (2 × 10^6^) and spheroids (~200) were separately mixed with matrigel as described in Material and Methods and injected subcutaneously (s.c.) into a dorso-caudal location of FVB/N male mice. A small, hard, well-shaped mass was noticed at the site of injection in each mouse ~10 days after spheroid injections and ~15–20 days after injection of cells (Supplementary Figure S2). At these times, the tumors derived from both the cells and spheroids exhibited well-delimited borders of ~5–7 mm in diameter. These data indicated that although both cells and spheroids had the ability to generate tumors, the tumors derived from spheroids arose more rapidly. Tumors derived from HMVP2 spheroids continued to grow to a maximum allowable limit of 15 mm diameter within 45 days. Figure 2D shows representative H & E sections of tumors derived from HMVP2 spheroids and Figure 2E shows that these tumors expressed Sca-1, CD49f, CK14 and CK8 (panels a,b,c,d, respectively in Figure 2E).

### Establishment of additional cell lines following recovery from allograft tumors

**Figure 3:**
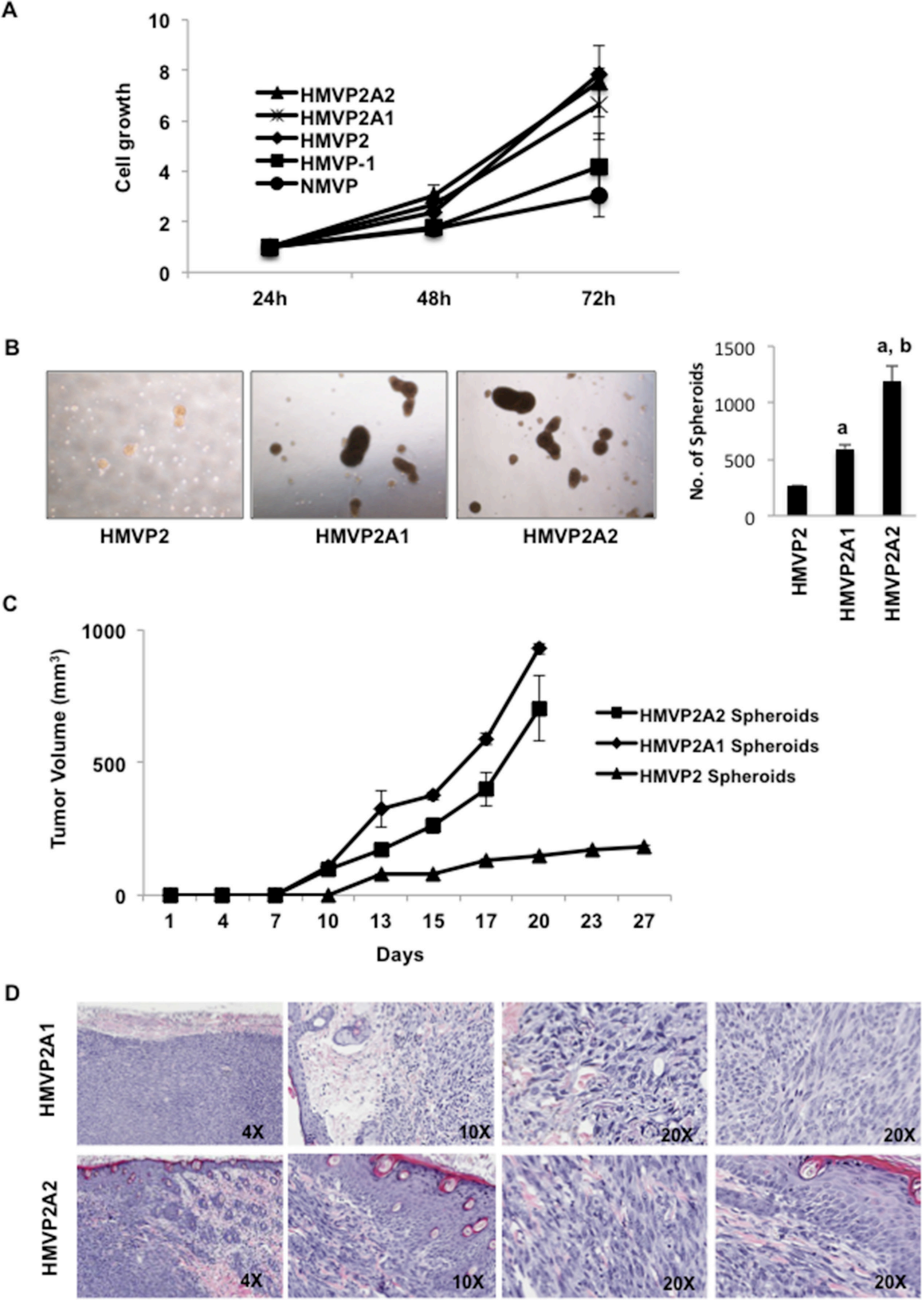
Further characterization of HMVP2A1 and HMVP2A2 cells (**A**) *In vitro* proliferation rate of NMVP, HMVP1, HMVP2, HMVP2A1 and HMVP2A2 cells. Cells were seeded in 96 well plates and the proliferation was measured at 24, 48 and 72 hrs. by MTT assay. Values are mean ± SEM of three independent experiments. (**B**) Comparison of size and number of spheroids from HMVP2, HMVP2A1 and HMVP2A2 cells after 72 hours of culture. a, significantly different (*P* < 0.01) from HMVP2 cells; and b, significantly different (*P* < 0.01) from HMVP2A1 cells. Values are mean ± SEM of three independent experiments. (**C**) Growth of allograft tumors derived from HMVP2, HMVP2A1 and HMVP2A2 spheroids in male FVB/N mice. Values represent mean ± SEM from tumors in 4 mice per group. (**D**) Representative H & E stained section of tumors, at both low and higher magnification, derived from HMVP2A1 (top panels) and HMVP2A2 (bottom panels) tumors.

Tumors generated following injection of HMVP2 spheroids were collected as described in Material and Methods and using the same protocol described for the original isolation for HMVP2 cells, an additional cell line was established. After 4–5 passages, a homogenous cell population was obtained. These cells, referred to as HMVP2A1 cells, also showed the ability to generate spheroids and develop tumors when injected into syngeneic FVB/N mice (see below). Furthermore, an additional cell line was derived from tumors generated following injection of HMVP2A1 cells using a similar procedure and these cells were referred to as HMVP2A2. A representative picture of all cell lines is shown in Supplementary Figure S3. Figure 3A shows the growth rate all of the cells lines developed in this study. HMVP2, HMVP2A1 and HMVP2A2 cells exhibited a higher proliferation rate than HMVP1 and NMVP cells. As shown in Figure 3B, HMVP2A1 and HMVP2A2 cells possessed the ability to form spheroids with a greater propensity than HMVP2 cells. In addition, tumors produced by injection of the HMVP2A1 and HMVP2A2 spheroids (~ 200 spheroids per injection) showed more rapid growth rates compared to the parental HMVP2 cells (Figure 3C). Histopathologic evaluation of the tumors confirmed undifferentiated masses containing a variety of cell types including epithelial-shaped cells, blood vessels, inflammatory cells and adipocytes (Figure 3D). Neovascularization and hemorrhage was also observed.

We also performed immunocytochemical (ICC) analysis using various antibodies against cytokeratins, epithelial and stem cell markers in all the cell lines. As shown in Supplementary Figures S4 and S5, the expression of epithelial cell markers CK5, CK14 and E-cadherin could be detected in all cell lines (See Supplementary Figure S4). In addition, the expression of various stem cell markers such as Sca-1, ALDH1 appeared to be higher in the more tumorigenic cell lines (see Supplementary Figure S5).

**Figure 4:**
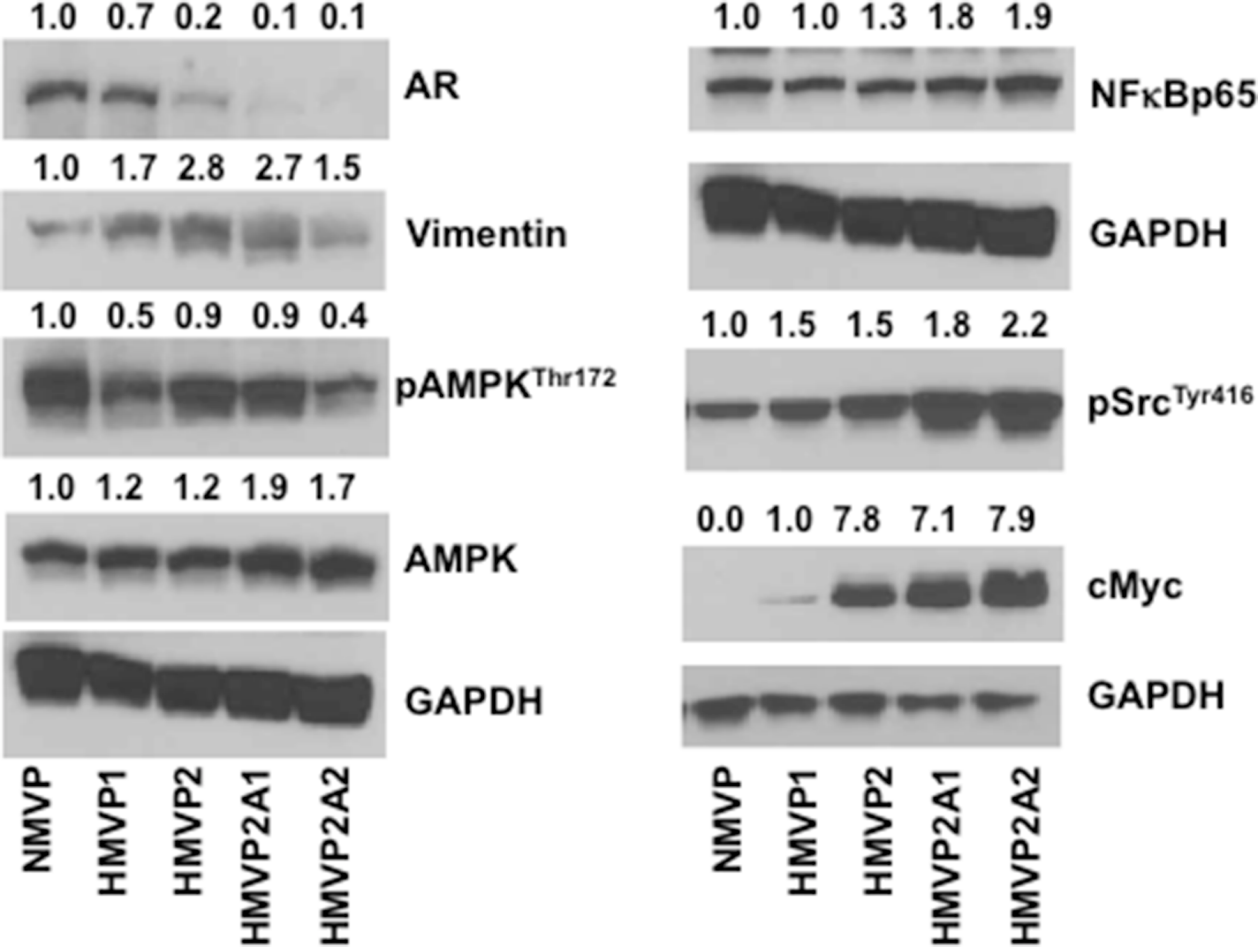
Western blot analyses of the protein lysates of all mouse cells cultured *in vitro* Cells were seeded in 100 mm culture dishes and allowed to grow until 60–70% confluence. Protein lysates were subjected to Western blot for AR, vimentin, pAMPK, AMPK, NFκBp65, pSrc and cMyc. GAPDH was used for protein loading control. Quantification value presented on top of each blot was normalized to GAPDH and total proteins where appropriate.

Western blot analyses of lysates from cells for selected proteins are shown in Figure 4. Expression of the androgen receptor (AR) was detected in NMVP and HMVP1 cell lines, however, expression of the AR was significantly lower in HMVP2 cells and almost absent in HMVP2A1 and HMVP2A2 cells. Phosphorylation of c-Src, and expression of NF-κB, c-Myc and vimentin was higher in HMVP2 and its derivative cell lines compared to both NMVP and HMVP1 cells.

### Androgen sensitivity of cell lines

**Figure 5:**
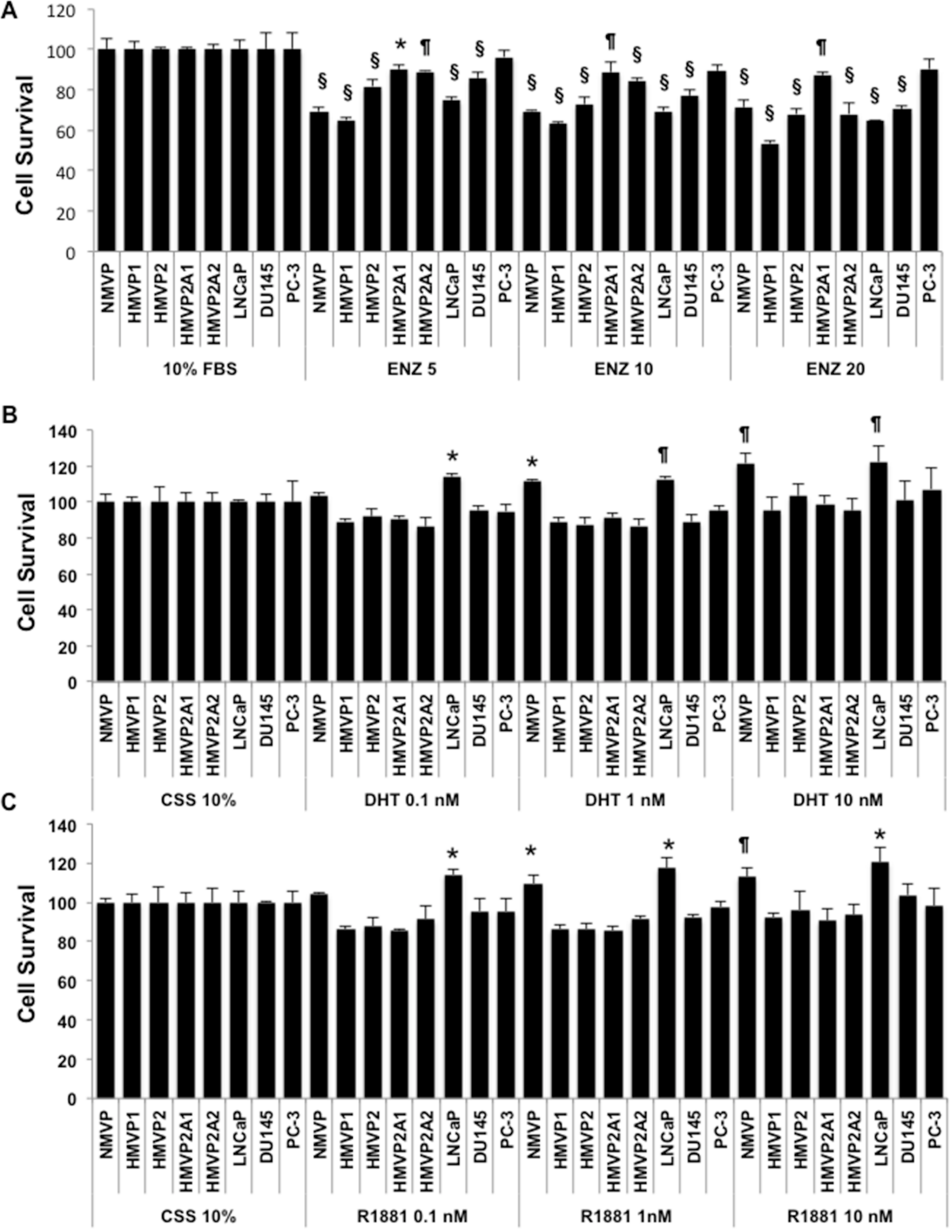
Androgen sensitivity of the cell lines Effect of androgens and anti-androgens on the growth/survival of isolated mouse prostate (normal and tumor) and human PCa cells. Cells (1–5 × 10^3^/well) were plated in 96 well tissue culture plates and treated with the indicated concentration of ENZ (**A**), dihydrotestosterone (DHT) (**B**) or R1881 (**C**) for 72 h and cell growth/survival was measured by MTT assay. The decrease in cell survival after treatment with ENZ was statistically significant for all cell lines except PC-3 cells measured by one-way ANOVA followed by Bonferroni's multiple comparison test (*P < 0.05, ^¶^*P* < 0.01 and ^§^*P* < 0.001 compared to control). The increase in cell survival was statistically significant as determined by one-way ANOVA followed by Bonferroni's multiple comparison test as indicated, *P < 0.05, ^¶^*P* < 0.01.

In light of the different AR protein levels in the various cell lines, the androgen sensitivity of these cell lines was investigated. As shown in Supplementary Figure S6, NMVP and HMVP1 cells grown in 10% charcoal stripped serum (CSS) containing media showed a significant decrease in growth. Growth of HMVP2 cells was also decreased in CSS containing media but to a lesser extent compared to NMVP and HMVP1 cells. Both HMVP2A1 and HMVP2A2 cells showed no change in cell growth in CSS containing media. We also used several human PCa cell lines such as LNCaP, DU145 and PC-3 cells and as expected the androgen dependent LNCaP cells were more susceptible to CSS mediated growth suppression than androgen independent DU145 and PC-3 cells (Supplementary Figure S6A and 6B). Next, we treated both human and mouse PCa cells with different concentrations of enzalutamide (ENZ). As shown in Figure 5A and Supplementary Figure S7, survival of NMVP, HMVP1 and HMVP2 cells was reduced to a greater extent by treatment with ENZ compared to HMVP2A1 and HMVP2A2 cells. This can be seen at the lower concentrations of ENZ. Among the human PCa cells, LNCaP cells showed the highest decrease in survival following treatment with ENZ. Finally, we examined the effect of dihydrotestosterone (DHT) and the synthetic testosterone analog R1881, on the growth properties of all the cell lines. As shown in Figure 5B and 5C, both dihydrotestosterone and R1881 increased the growth of NMVP and LNCaP cells. In contrast, no significant alteration in growth of the other cell lines was observed when treated with androgens under the experimental conditions employed.

## DISCUSSION

Conventional therapies for PCa rely on removing or blocking the actions of androgens resulting in regression of the cancer initially due to the requirement of androgens for cancer cell growth and survival (reviewed in [31, 32]). Unfortunately, a significant proportion of treated cancers eventually relapse becoming androgen independent and are no longer responsive to conventional therapy [33, 34]. PCSCs are postulated to play a key role in tumor survival and recurrence although this remains controversial [35, 36]. Mouse models have proven extremely useful for studies of PCa development and progression [37–40]. In the present study, we have developed cell lines derived from tumors arising in the Hi-Myc mouse model of PCa [23] to allow further analyses of the mechanisms of tumor development in this mouse model and to provide tools for further preclinical studies.

Using previously described protocols for the specific isolation of progenitor epithelial cells [3, 5, 17–21, 24–26], we isolated and established a cell line from the VP of one year old Hi-Myc mice characterized as LIN^neg^/Sca-1^high^/CD49f^high^. These cells, called HMVP2, exhibited a series of *in vitro* and *in vivo* characteristics attributed to PCSCs [13–15, 18, 21]. In culture, HMVP2 cells showed a high proliferation rate when compared to cell lines established from the VP of wild-type FVB/N mice and 6 month old Hi-Myc mice. HMVP2 cells also readily formed spheroids. Furthermore, cells obtained from spheroids could be repeatedly regenerated. HMVP2 spheroids were initially characterized as Lin^neg^ Sca-1^high^ CD49f^high^ and also stained positive for Sca-1, CK14 and CK8. CK8 stained cells were primarily located in the periphery of the early spheroids. Thus, the majority of the cells in early spheroids were of a basal-epithelial character similar to the original HMVP2 cells. In mouse and human prostate, basal cells can be considered the stem cells of the glands and they differentiate into luminal cells, having a more specific secretory function [12]. Notably, HMVP2 spheroids underwent a time dependent differentiation from basal-epithelial (Lin^neg^ Sca-1^high^ CD49f^high^) type to luminal-epithelial cell type (Lin^neg^ Sca-1^low^ CD49f^low^) when compared to cells grown in monolayer cultures. It is established that spheroid structures or 3-D cell culture allows cell-to-cell interaction that closely resembles cell-cell interactions *in vivo* when compared to cells in conventional monolayer culture [41, 42]. Nevertheless, there remained a population of cells expressing the basal-epithelial Lin^neg^ Sca-1^high^ CD49f^high^ phenotype even after 4 days. Moreover, a range of cell subpopulations expressing CD34, CD44 and CD29 markers gated on the two cell subpopulations, LIN^neg^ Sca-1^high^ CD49f^high^ and Lin^neg^ Sca-1^low^ CD49f^low^, was detected upon additional FC analyses. The level of these markers fluctuated in the two cell subpopulations suggesting a differentiation from the original HMVP2 cells although more functional analyses are required in order to fully validate these initial findings. Collectively, these data support the hypothesis that HMVP2 cells have the capacity to differentiate into multiple cell types and possess stem-like characteristics.

Cells isolated from HMVP2 spheroids were able to regenerate spheroids repeatedly further demonstrating the stem-like property of these cells. In addition, both HMVP2 cells and spheroids were capable of initiating tumors in syngeneic mice, although tumors developed more rapidly from spheroids. Rapidly growing tumors derived from spheroids invaded the underlying muscle layer within the time period of the experiments. The tumors derived from HMVP2 spheroids retained characteristics of the initial cell population as well as more differentiated cell populations as assessed by IF staining of Sca-1, CD49f, CK14 and CK8. As part of this study, additional cell lines were generated by isolating tumors derived from HMVP2 spheroids and culturing these cells (HMVP2A1 cells) followed by injection of spheroids from these cells and culturing of the isolated tumor cells (HMVP2A2 cells). Both of these additional cell lines possessed greater propensity to generate spheroids suggesting greater stem-like properties compared to the parental HMVP2 cells. IF staining (See Supplementary Figure S5) further confirmed this observation, as the expression of the stem cell markers Sca-1 and ALDH1 appeared to be higher in both HMVP1A1 and HMVP2A2 cells compared to the other cell lines. These additional cell lines readily produced tumors in syngeneic mice with a rapid growth rate.

Western blot analyses of all the cell lines generated as part of this study (Figure 4) revealed that the AR was expressed in NMVP and HMVP1 cells and to a lesser extent in HMVP2 cells. Interestingly, the level of AR protein was significantly decreased and almost undetectable in both HMVP2A1 and HMVP2A2 cells. Treatment of cells with ENZ led to a significant decrease in NMVP, HMVP1 and HMVP2 cell survival at all concentrations. Survival of HMVP2A1 and HMVP2A2 cells that expressed low levels of the AR was not affected to as great an extent by ENZ, especially HMVP2A1 cells. Addition of androgen to the culture media further increased the growth of NMVP cells but had no significant effect on growth of other mouse PCa cell lines under the culture conditions employed. These data suggest that NMVP cells were the most androgen dependent cells and that the other PCa cells derived from Hi-Myc mice were less dependent on androgens for maximum growth/survival. HMVP2A1 cells appeared to be the least androgen dependent of the PCa cell lines. Further work will be required to fully evaluate the androgen sensitivity of these cell lines.

Phosphorylation of Src was less in NMVP cells and gradually higher in cells derived from HiMyc mice. It is now well established that activation of Src is common in many cancers (reviewed in [43]). Src can function as an upstream and downstream modulator of various receptors and non-receptor molecules including various growth factors such as epidermal growth factor receptor (EGFR), JAK-STAT, PI3K/Akt and mitogen activated protein kinase (MAPK) signaling leading to regulation of cell cycle, survival and proliferation [43]. NFκB signaling is also involved in various human cancers including PCa [44]. In the present study, the increased growth properties of HMVP2A1 and HMVP2A2 cells compared to the other cell lines might be due in part, to increased expression of pSrc and NFκB. AMPK is another important energy sensing signaling molecule that modulates various signaling events including mTORC1 signaling involved in proliferation process [45]. We found decreased phosphorylation of AMPK in HMVP1 and HMVP2A2 cells but not in HMVP2 and HMVP2A1 cells. Additional functional analyses will be needed to fully understand the exact role of pAMPK in terms of the growth properties of these newly established cell lines. Finally, we also found increased expression of c-Myc in HMVP2, HMVP2A1 and HMVP2A2 cells. Increased c-Myc expression in these cell lines may have also contributed to the more rapid cell growth and tumorigenic potential. Since the antibody used in the current study detects both endogenous (mouse) and human (transgene) c-Myc, the mechanism for increased c-Myc expression in these cell lines remains to be determined.

Expression of vimentin was lower in NMVP cells compared to other cell lines. Vimentin is one of the most widely expressed and highly conserved proteins of the type III intermediate filament protein family. Vimentin is expressed during the process of epithelial-mesenchymal transition (EMT) [46]. During the reverse process of EMT, known as mesenchymal-epithelial transition (MET), cells start acquiring epithelial characteristics and show a decreased vimentin expression with lower cell motility rates [47]. Increased vimentin expression has been reported in various tumor cell lines and tissues including prostate cancer [48–54]. In many cancers, vimentin overexpression correlates well with accelerated tumor growth, invasion, and poor prognosis [46]. However, the exact role of vimentin in cancer progression remains to be fully elucidated. The high level of vimentin expression in the Hi-Myc derived tumor cell lines may allow further dissection of its role in tumor growth and progression. In addition, ICC staining showed a gradual decrease in epithelial markers such as CK5, CK14 and E-Cadherin from NMVP to HMVP2A2 cells further suggesting an EMT process in the more tumorigenic cell lines.

In conclusion, we have developed cell lines derived from tumors that developed in Hi-Myc mice with progenitor/stem-like characteristics and tumor initiating ability. These cells will be invaluable for future mechanistic studies of PCa tumor development. In addition, these cells will provide a valuable model system for testing novel agents for both the prevention and treatment of PCa. In this regard, we recently completed a study to evaluate a compound found in dried ginger, 6-shogaol, for its ability to inhibit growth of HMVP2 cells in culture as well as inhibit the growth of tumors initiated by injection of HMVP2 spheroids *in vivo* demonstrating the utility of these cells for preclinical studies [55]. Further characterization of these cells and the other cell lines established as part of this study is ongoing.

## MATERIALS AND METHODS

### Reagents

RPMI-1640 and fetal bovine serum (FBS) were obtained from Life Technologies. Dispase, Balanced Salt Solution (HBSS), DNase I, collagenase/Hyaluronidase were from Stem Cell Technologies. Antibodies against AMPK, pAMPK^Thr172^, NFκBp65, pSrc, Sca-1, CD49f, cMyc, and GAPDH were purchased from Cell Signaling. Antibodies for Sca-1, CD49f, CD34, CD44, CD29, CD31, CD45 and Ter119 used for FC analysis were from eBioscience. ALDH1 antibody was purchased from Abcam. Antibodies for CK5, CK8, CK14 were purchased from SantaCruz Biotechnologies.

### Cell culture

LNCaP, DU145 and PC-3 cells were purchased from the ATCC. These cells were maintained in RPMI-1640 medium with 10% FBS. Cell lines were authenticated by genetic biomarkers. Mycoplasma test was performed by PCR amplification (Applied Biological Materials Inc.) and 4′,6-diamidino-2-phenylindole (DAPI) staining. All cells were cultured in 95% air and 5% CO2 at 37°C.

### Isolation and establishment of cell lines from the prostate of FVB/N and Hi-Myc mice

Several methods have been described for the isolation of primary cells from the mouse prostate [3, 56–58]. In this study, cells were isolated using a protocol from Stem Cells Technologies (Vancouver, Canada). VP glands from wild-type FVB/N mice and six month old and one-year-old male Hi-Myc mice were isolated and dissected (*N* = 5). In a Petri dish, VP tissues were washed with cold PBS, and mechanically disaggregated with surgical blades. Dispersed prostate tissues were then immersed in a collagenase/hyaluronidase mixture with 9 parts DMEM/F12 supplemented with 5% FBS, placed into a 15 ml centrifuge tube and then incubated for 3 hours at 37°C. After dissociation, cells were centrifuged at 350 *g* for 5 minutes with the brake on and the supernatant was discarded. The pellet was resuspended in 5 ml of 0.25% Trypsin-EDTA and placed on ice for 1 hour. Ten mL of cold HBSS supplemented with 2% FBS was added, and then the cells were centrifuged at 350 *g* for 5 minutes with the brake on. The supernatant was removed and 2 ml of pre-warmed 5 mg/mL dispase and 200 μl of 1 mg/ml DNase I was added. Samples were pipetted for 1 minute to resuspend the cells and then 10 ml of cold HBSS supplemented with 2% FBS was added. Cell suspensions were filtered through a 40 μm cell strainer and then centrifuged at 350 *g* for 5 minutes with the brake on to prevent dissociation of the cell pellet. The resultant cell pellets were resuspended in RPMI/10%FBS medium for subsequent assays. A similar protocol was used to generate NMVP cells (from wild-type FVB/N mice), HMVP1 cells (derived from the VP of six months old Hi-Myc mice) and for the HMVP2A1 (derived from HMVP2 allograft tumors) and HMVP2A2 cells (derived from HMVP2A1 allograft tumors).

### Generation of spheroids and re-regeneration assays

For the generation of spheroids, HMVP2 cells from a monolayer culture were washed twice with PBS, trypsinized, dissociated and seeded at a concentration of 2 × 10^4^ cells/ml in 100 mm ultra-low adherent dishes with RPMI/10% FBS media. Spheroid formation was observed from day one with a consistent linear growth in shape and number up to day 5. For re-regeneration assays, spheroids on day four of culture were washed in PBS, trypsinized, gently dissociated, filtered through a 40 μm strainer and cells were re-seeded at a concentration of 2 × 10^4^ cells/ml in 100 mm ultra-low adherent dishes with RPMI/10% FBS medium.

### Proliferation assays

The proliferation of cells was measured by MTT assay as previously described [59]. Briefly, cells (2–4 × 10^4^/mL) were plated in 96-well plate. After 24, 48 and 72 hours of incubation, the cells were washed with fresh medium, treated with MTT solution, and incubated for an additional 3 hours. The formazan crystal was dissolved in 100 μL SDS solution, added to the cells and the absorbance was measured at 570 nm using Tecan infinite 200 pro microplate reader (Tecan System Inc.).

### *In vivo* tumor development in syngeneic mice

HMVP2 cells and spheroids were cultured for three days. Following trypsinization, cells were washed twice, re-suspended in a concentration of 2 × 10^6^ cells mixed with 200 μl 45% RPMI medium + 5% FBS + 50% matrigel membrane matrix (BD) and then the mixture was injected subcutaneously (SC) in a dorso-caudal location of two month old male FVB/N mice (*N* = 10). Mice were slightly sedated with isofluorane prior to injection. Similarly, approximately 200 spheroids resulting from an initial culture of 2 × 10^5^ cells were washed and re-suspended in fresh 45% RPMI medium + 5% FBS + 50% matrigel (200 μl total volume) and then injected subcutaneously (SC) in a second set of mice (*N* = 10). Mice were observed daily for tumor formation and small SC masses (~5 mm in diameter) were seen after two weeks of injection. Tumor masses were then measured twice a week with a digital caliper and tumor volume was calculated using the formula, tumor volume = ½ length × width^2^ [60].

### Flow cytometry (FC) assays

VP tissues from non-transgenic FVB/N mice (control) and one-year-old Hi-Myc transgenic mice were dissected and cells were isolated using the progenitor cell isolation procedure described above. Harvested cells were then passed through a 40 μm nylon mesh and then resuspended in PBS containing 1% BSA and 2 mM EDTA (FACS buffer). FcR blocking reagent (Miltenyi Biotec) was added at 1:100 for 15 minutes at 4°C prior to staining. Antibody staining was performed in FACS buffer. Live cells were distinguished from dead cells by gating using the forward and side scatter signals as well as by exclusion of 7-amino actinomycin D (eBioscience, USA). Non-stained cells or appropriate isotype control-stained cells were used to evaluate background fluorescence. Cells were then labeled with a combination of monoclonal antibodies against Sca-1, CD49f, CD34, CD44, CD29, CD31, CD45 and Ter119 for 30 minutes at 4°C. All samples were analyzed on a BD FACS AriaII (BD Biosciences). Data was analyzed using FlowJo Software (Version 7.2.5, Tree Star, Ashland, OR).

### Histological, immunohistochemical (IHC) and immunocytochemical (ICC) analyses

For histological analysis, spheroids embedded in 50% gelatin-50% media and tumors were fixed in formalin prior to sectioning. Sections of 5 μm were cut and stained with hematoxylin and eosin (H & E). The expression and localization of the target proteins in cultured cells, spheroids, and tumor tissue were determined using IF staining on sections as described previously [55, 61]. Briefly, cells cultured on a chamber slide (Lab-Tek 4 chamber system slide, Lab-Tek) were fixed with 4% paraformaldehyde in PBS for 15 min. The sections of spheroid and tumor were deparaffinized prior to IF staining. The sections of cultured cells, spheroids and tumor were processed for IF as follows; after blocking by 10% goat serum in 1% Tween 20/TBS (Tris-buffered saline) for 60 min, the cells were incubated with primary antibodies for overnight at 4°C followed by incubation with specific fluorescent-conjugated secondary antibodies for 45 min. Sections were analyzed using a laser confocal microscope (Leica SP5).

### Western blot analyses

Fifty μg of cell protein lysates were electrophoretically separated on a 4–15% SDS-PAGE gel and transferred onto nitrocellulose membrane (BioRad Laboratories, Hercules, CA) [55]. After blocking with 5% BSA, the membranes were incubated with primary antibodies. After incubation with corresponding secondary antibodies, proteins were visualized using a commercial chemiluminescent detection kit (Thermo Scientific).

### Statistical analyses

Statistical analyses were conducted using either the Student's *t* or One Way ANOVA followed by Bonferroni's multiple comparison test as indicated. Significance was set at *P* ≤ 0.05 in all cases.

## SUPPLEMENTARY MATERIALS FIGURES AND TABLES


