# Supplementary material for: Linneg Sca-1high CD49fhigh prostate cancer cells derived from the Hi-Myc mouse model are tumor-initiating cells with basal-epithelial characteristics and differentiation potential in vitro and in vivo
Source: Oncotarget. 2016 Feb 20;7(18):25194–207. doi: 10.18632/oncotarget.7535 (PMC5041897; doi:10.18632/oncotarget.7535)
Supplement: Supplementary file 1 [file oncotarget-07-25194-s001.pdf]

Lin^{neg} Sca-1^{high}CD49f ^{high} prostate cancer cells derived from the Hi-Myc mouse model are tumor-initiating cells with basal-epithelial characteristics and differentiation potential *in vitro* and *in vivo*

Supplementary Materials

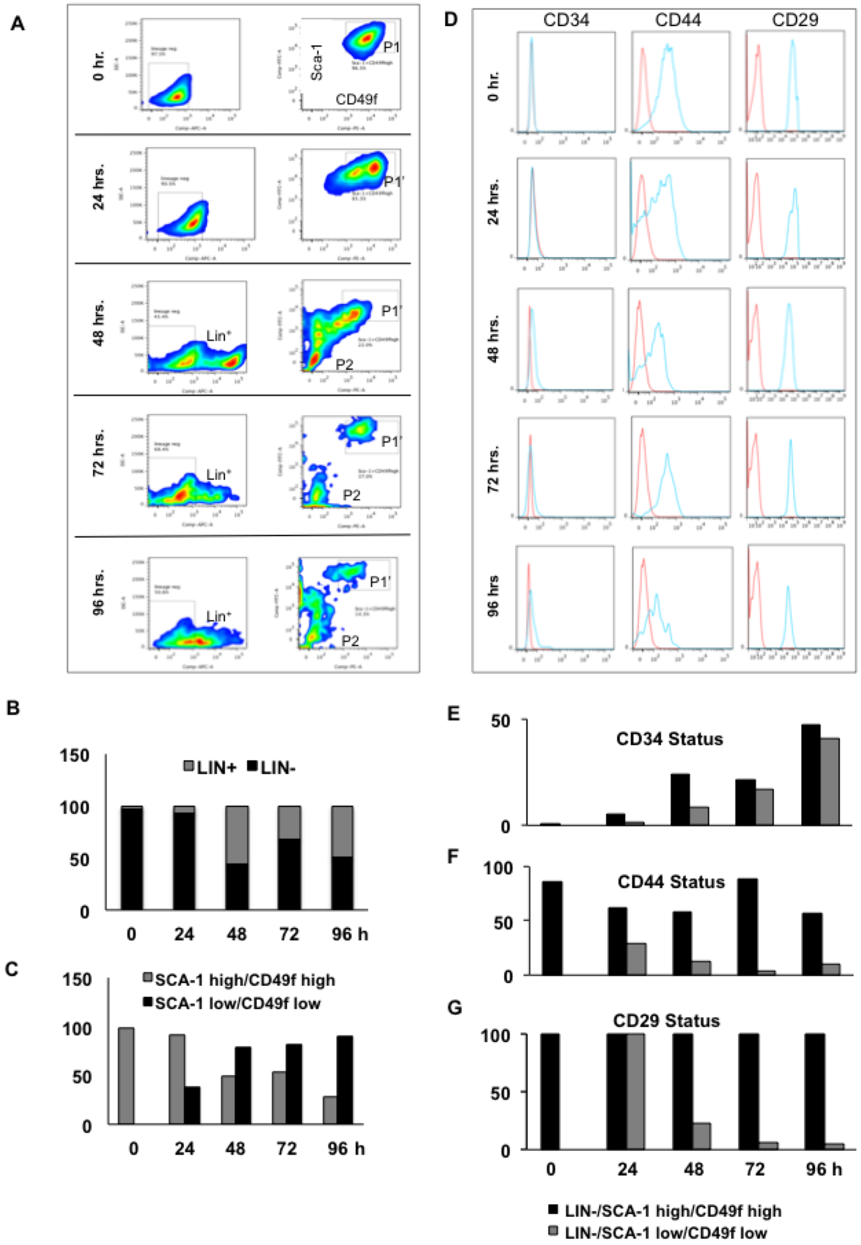

Supplementary Figure S1: Analysis of spheroid cell populations over time. HMVP-2 cells were seeded for spheroid formation and were stained by FC at 0, 24, 48, 72 and 96 hrs**.** (**A**) At the time of seeding, a single homogenous cell type expressing only Lin^{neg} Sca-1^{high} CD49f ^{high} markers was detected (**A**) 0 hr. On the following subsequent days, cells collected from the spheroids showed a gradual differentiation to at least three cell subpopulations, Lin^{pos} cells, Lin^{neg} Sca-1^{high} CD49f^{high} cells and Lin^{neg}Sca-1^{low}CD49f^{low} cells (A 24 to 96 hrs, **B** and **C**). (**D**) FC analyses for CD34, CD44 and CD29 expression gated on Lin^{neg}Sca-1^{high} CD49f^{high} and Lin^{neg}Sca-1^{low}CD49f^{low} populations.

In this experiment, CD34 was absent in both the Lin^{neg} Sca-1^{high} CD49f^{high} cells at 0 hr, and in the differentiated Lin^{neg} Sca-1^{low} CD49f^{low} cells a day later, although expression gradually started to increase in both cell sub-populations up to almost 40–50% after 96 hrs in culture (D left column and **E**). CD44 was initially expressed in a large part of the original HMVP-2 Lin^{neg}Sca-1^{high} CD49f^{high} cells (about 90%) and the expression was lower on the Lin^{neg} Sca-1^{low} CD49f^{low} sub-population after one day of culture (~ 30%). CD44 expression was gradually decreased in both cell populations to ~ 60% at 96 hrs in the cells expressing Lin^{neg} Sca-1^{high} CD49f^{high} suggesting a loss of pluripotentiality. CD44 expression was almost completely lost in the Lin^{neg} Sca-1^{low} CD49f^{low} expressing cells, implying further cell differentiation (D middle column and **F**). CD29 was expressed throughout the four day assay period and was decreased in the Lin^{neg} Sca-1^{low} CD49f^{low} cells showing signs of further differentiation (D right column and **G**).

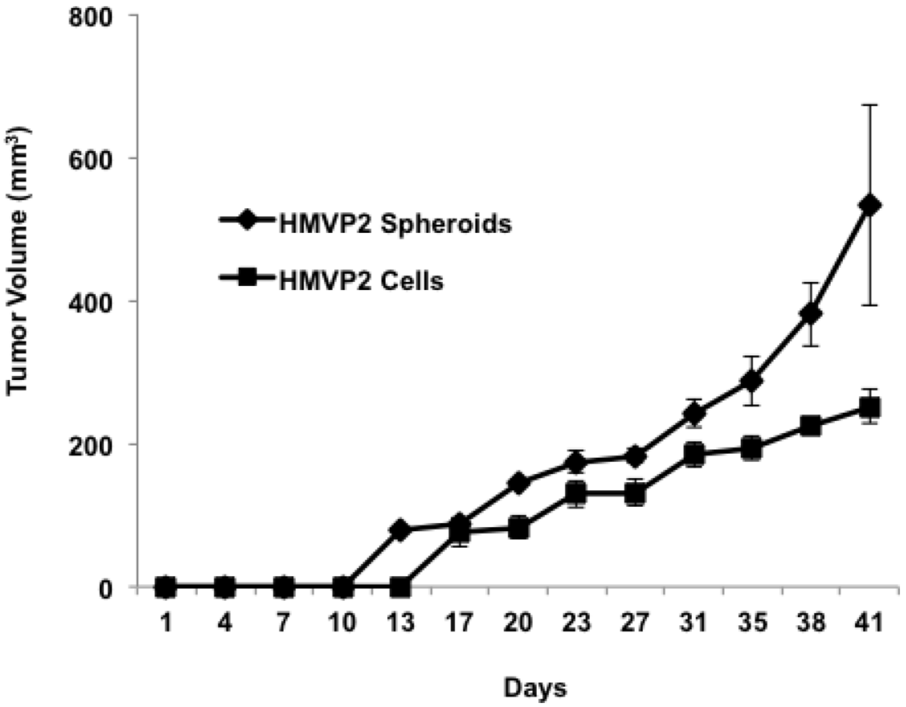

Supplementary Figure S2: Comparison of HMVP2 cells and HMVP2 spheroids for tumor growth in male FVB/N mice. Values represent mean ± SEM from tumors in 4 mice per group.

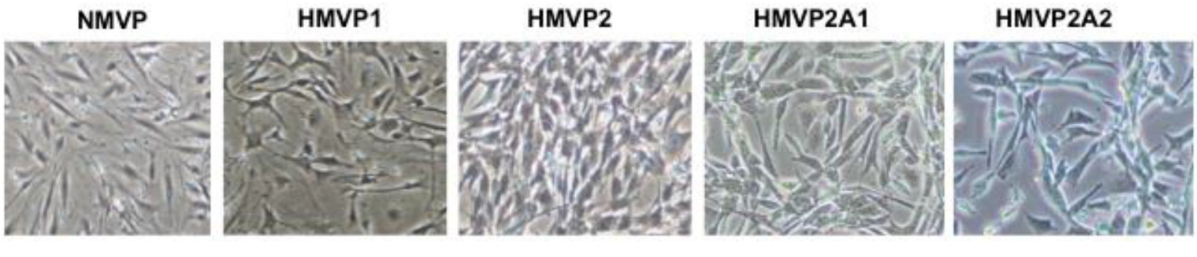

Supplementary Figure S3: Representative morphology of all established cell lines.

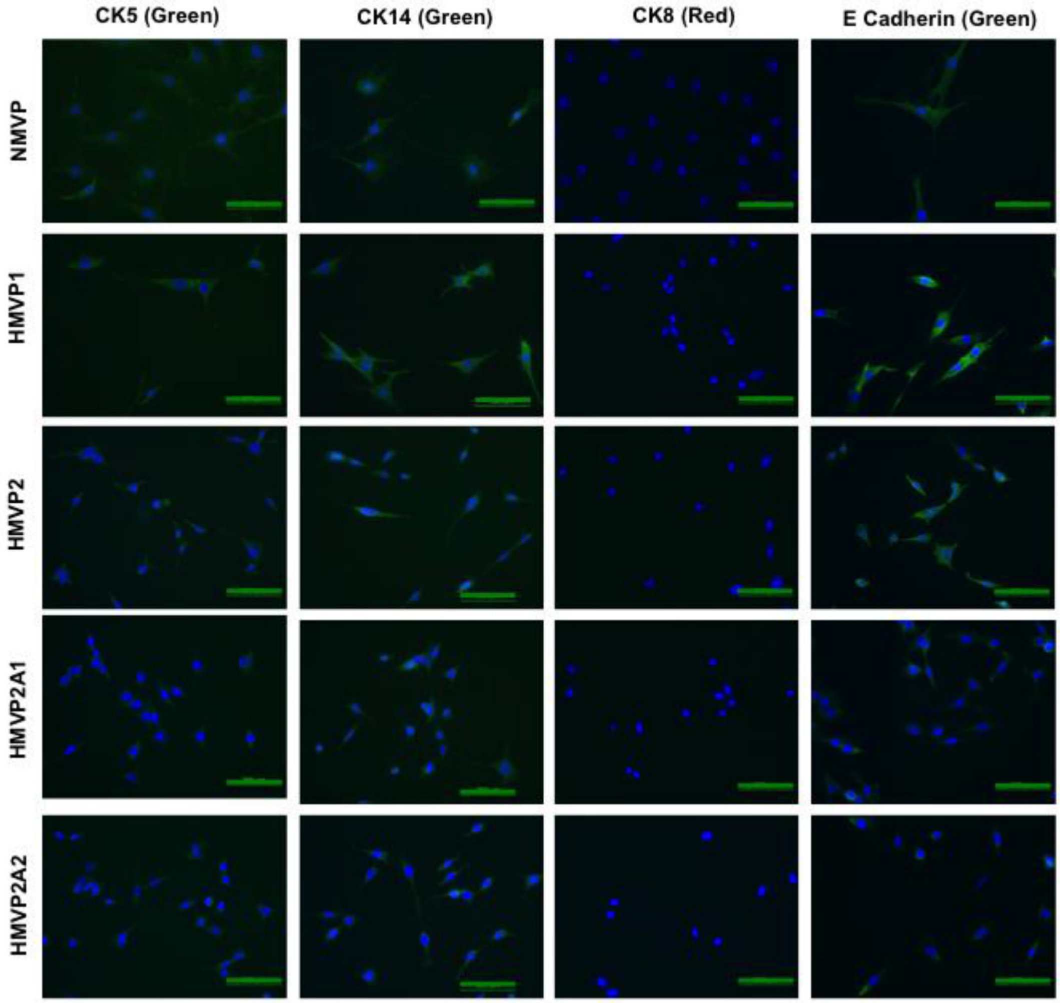

Supplementary Figure S4: ICC staining of NMVP, HMVP1, HMVP2, HMVP2A1 and HMVP2A2 cells for epithelial cell markers. Cells were allowed to grow in tissue culture inserts for 24 h and then stained for CK5, CK14, CK8 and E-cadherin (left to right). (The length of bar is 200 μm).

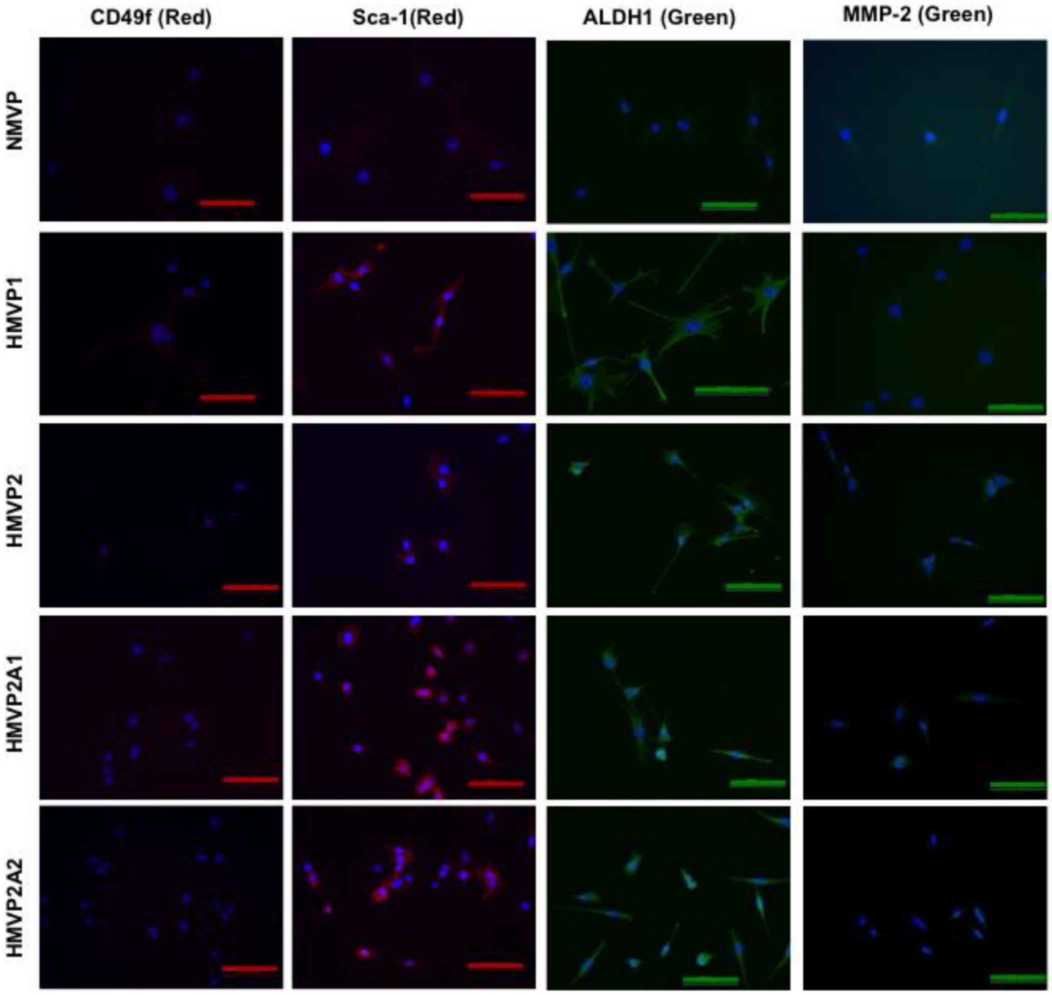

Supplementary Figure S5: ICC staining of NMVP, HMVP1, HMVP2, HMVP2A1 and HMVP2A2 cells for stem cell markers and MMP-2. Cells were allowed to grow in tissue culture inserts for 24 h and then stained for CD49f, Sca-1, ALDH1 and MMP-2 (left to right). (The length of bar is 200 μm).

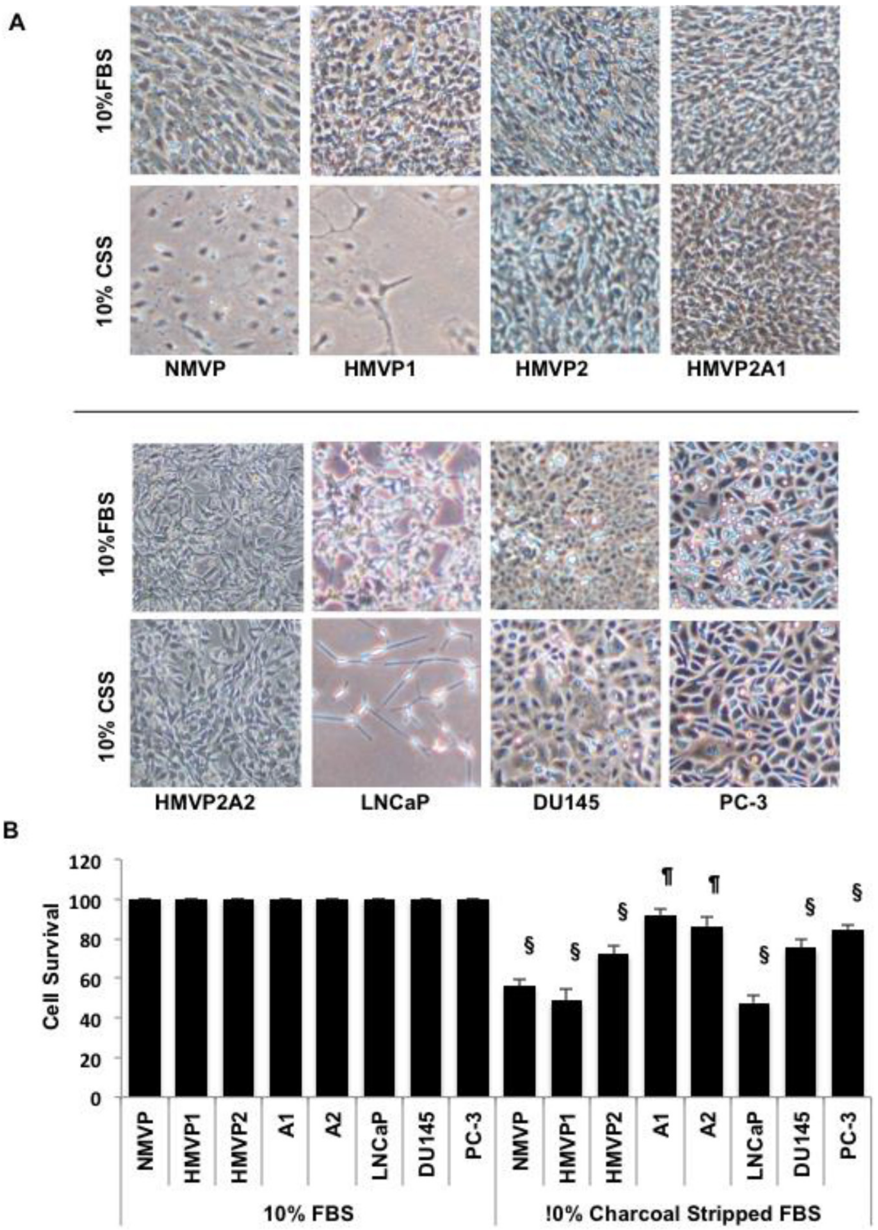

Supplementary Figure S6: Effect of charcoal stripped serum on the growth/survival of isolated mouse prostate (normal and tumor) cells and human PCa cells. Cells were plated in tissue culture dishes and the next day media was replaced with either 10% FBS or 10% charcoal stripped serum for 72 h. (**A**) Appearance of cells. Magnification is 20×. (**B**) cell growth/survival was measured by MTT assay. Significantly different ($^{¶}P < 0.01$, $^{§}P < 0.001$) from the respective control groups (10% FBS) as analyzed by Student's *t* test.

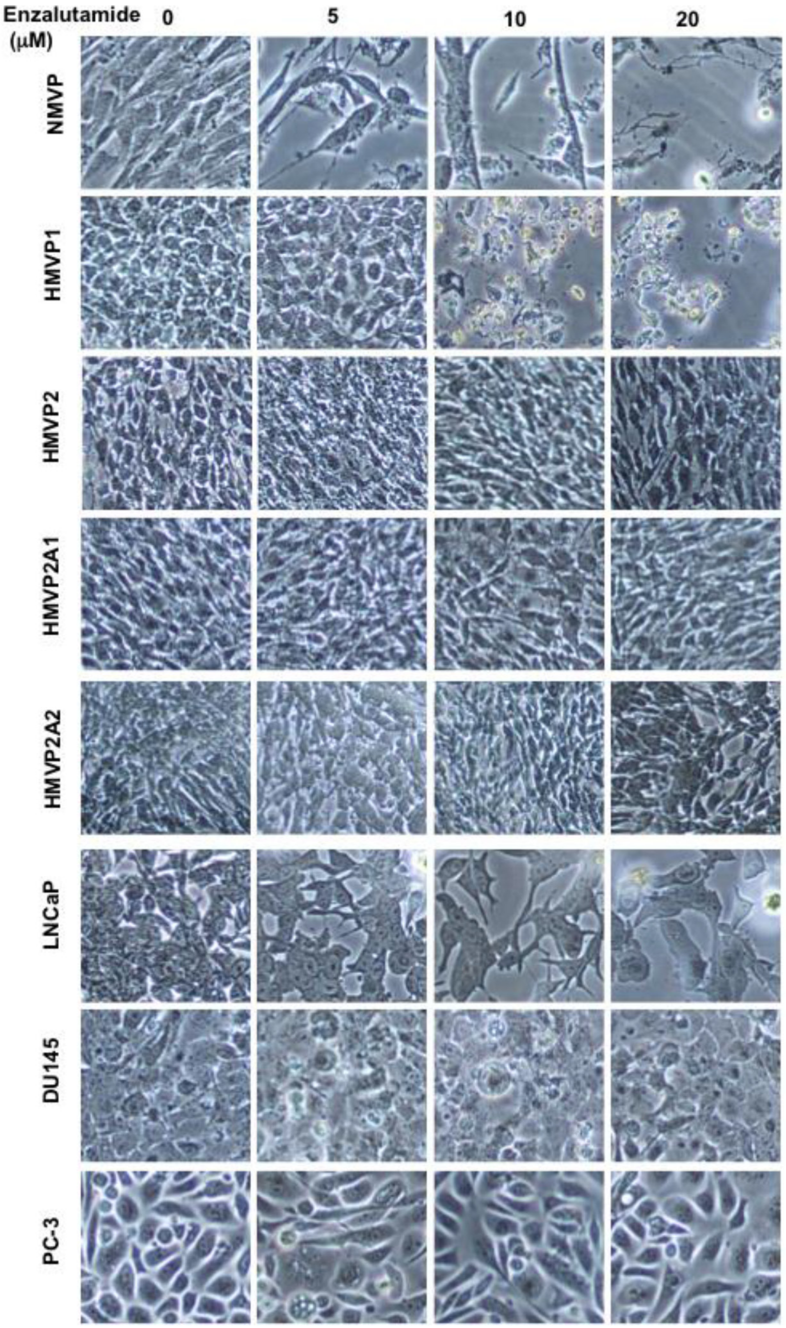

Supplementary Figure S7: Effect of enzalutamide (ENZ) on the growth/survival of isolated mouse prostate (normal and tumor) cells and human PCa cells. Cells were plated in tissue culture plates and treated with the indicated concentration of ENZ for 72 h. Magnification is 40×.
